# Effect of dural inflammatory soup application on activation and sensitization markers in the caudal trigeminal nucleus of the rat and the modulatory effects of sumatriptan and kynurenic acid

**DOI:** 10.1186/s10194-021-01229-3

**Published:** 2021-03-31

**Authors:** Eleonóra Spekker, Klaudia Flóra Laborc, Zsuzsanna Bohár, Gábor Nagy-Grócz, Annamária Fejes-Szabó, Mónika Szűcs, László Vécsei, Árpád Párdutz

**Affiliations:** 1 Department of Neurology, Interdisciplinary Excellence Center, Faculty of Medicine, Albert Szent-Györgyi Clinical Center, University of Szeged, Semmelweis utca 6, Szeged, H-6725 Hungary; 2 MTA-SZTE Neuroscience Research Group, Szeged, Hungary; 3 Faculty of Health Sciences and Social Studies, University of Szeged, Szeged, Hungary; 4 Department of Medical Physics and Informatics, University of Szeged, Szeged, Hungary

**Keywords:** Migraine, Trigeminal system, Inflammatory soup, Sumatriptan, Kynurenic acid, CGRP, TRPV1, nNOS

## Abstract

**Background:**

The topical inflammatory soup can model the inflammation of the dura mater causing hypersensitivity and activation of the trigeminal system, a phenomenon present in migraineurs. Calcitonin gene-related peptide, transient receptor potential vanilloid-1 receptor, and neuronal nitric oxide synthase are important in the sensitization process there.

5-HT_1B/1D_ receptor agonists, triptans are used as a treatment of migraine. Kynurenic acid an NMDA antagonist can act on structures involved in trigeminal activation.

**Aim:**

We investigated the effect of inflammatory soup induced dural inflammation on the calcitonin gene-related peptide, transient receptor potential vanilloid-1 receptor, and neuronal nitric oxide synthase levels in the caudal trigeminal nucleus. We also tested whether pretreatment with a well-known antimigraine drug, such as sumatriptan and kynurenic acid, a compound with a different mechanism of action, can affect these changes and if their modulatory effects are comparable.

**Material and methods:**

After subcutaneous sumatriptan or intraperitoneal kynurenic acid the dura mater of adult male Sprague-Dawley rats (*n* = 72) was treated with inflammatory soup or its vehicle (synthetic interstitial fluid). Two and a half or four hours later perfusion was performed and the caudal trigeminal nucleus was removed for immunohistochemistry.

**Results and conclusion:**

Inflammatory soup increased calcitonin gene-related peptide, transient receptor potential vanilloid-1 receptor, and neuronal nitric oxide synthase in the caudal trigeminal nucleus compared to placebo, which was attenuated by sumatriptan and kynurenic acid. This suggests the involvement of 5-HT_1B/1D_ and NMDA receptors in neurogenic inflammation development of the dura and thus in migraine attacks.

## Article highlights or key findings

Inflammatory soup can cause an increase in the CGRP, TRPV1, and nNOS levels in the TNC.

Sumatriptan was able to mitigate the effect of inflammatory soup.

Kynurenic acid could modulate the effect of inflammatory soup.

## Introduction

Migraine is a common condition affecting up to 15% of the world’s population [1]. The pathomechanism of this disorder is not fully understood, but the sterile neurogenic inflammation of the dura mater and the activation and sensitization of the trigeminal system (TS) play a crucial role in the attack [2].

In animal models, stimulation of trigeminal nerves causes a release of neuropeptides, resulting in meningeal blood vessel dilation, plasma extravasation, platelet activation, and mast cell degranulation characteristic of neurogenic inflammation [3]. Calcitonin gene-related peptide (CGRP) is a multifunctional regulatory neuropeptide [4] and a key player in migraine: Serum concentrations of CGRP are elevated during the attack [5] whereas intravenous infusion of CGRP can induce a migraine-like headache in migraineurs [6]. In a rat model of migraine, electrical stimulation of the trigeminal ganglion was able to increase blood flow on the same side of the facial skin which was reduced by intravenous administration of the CGRP antagonist, CGRP 8–37 [7]. Transcranial electrical stimulation in rats was able to cause CGRP release and vasodilation, which was prevented by olcegepant, a CGRP receptor blocker [8]. These results are in line with the clinical data showing that CGRP antagonists are effective in the acute treatment of this disease [9–11]. On the other hand, there are other pathways involved in neurogenic inflammation which are not directly related to CGRP e.g. the appearance of cortical spreading depression might also contribute to this phenomenon [12].

Transient receptor potential vanilloid-1 receptor (TRPV1), a nonselective cation channel, a molecular component of pain detection and modulation [13], is selectively expressed by small- to medium-diameter neurons within the dorsal root ganglion (DRG) and trigeminal ganglia (TG), co-localized with CGRP in the latter [14]. TRPV1 activation leads to the release of neuropeptides, such as substance P and CGRP. These neuropeptides cause vasodilation and initiate neurogenic inflammation within the meninges under experimental conditions [15].

The synthesis of nitric oxide (NO) is catalyzed by neuronal nitric oxide synthase (nNOS), which can be found in the superficial layers of the dorsal horn of the spinal cord underlining its importance in the trigeminal pain processing [16]. Furthermore, its presence is confirmed in dural mast cells, trigeminal nerve endings, and Gasserian ganglion cells [17]. Systemic administration of nitroglycerin (NTG), a nitric oxide donor can induce an immediate headache and in migraine patients, this is followed by a typical migraine attack without aura [18]. The immediate headache can be explained by the vasodilatory effect of NO, which activates the dural nociceptors, while the delayed headache might be mainly due to an effect of NO on central nociceptors, causing a long-lasting endogenous synthesis of NO by enhancing nNOS resulting in central sensitization process [19].

Triptans are used to relieve migraine attacks, being an agonist on 5-hydroxytryptamine receptors (5-HT_1B/1D_), they can cause the constriction of dilated cranial arteries and the inhibition of CGRP release [20]. They block the depolarization of the trigeminal nerves and inhibit the neurotransmission at the level of interneurons of TNC [21]. In an animal model of migraine, after creating neurogenic inflammation in the dura mater, triptans were able to reduce the plasma protein extravasation, probably by inhibiting nociceptor activation and preventing neuropeptide release [5]. The increase of jugular vein CGRP concentration after the stimulation of the TG can be reduced by sumatriptan [5, 22]. In the rat model of trigeminal neuropathic pain, triptans can selectively inhibit nociceptive [23] and neuropathic pain behavior [24] and evoked activity in trigeminal dorsal horn neurons [21] in response to noxious stimulation of the trigeminal nerve area. In the TG, 5-HT_1B/1D_ receptors and glutamate were co-localized in several neurons [25], thus triptans may modulate glutamate release from trigeminal neurons through the 5-HT_1B/1D_ receptors and possibly reduce pain [24].

Kynurenic acid (KYNA) is a neuroactive product of the kynurenine pathway of tryptophan metabolism, which can exert its effect through N-methyl-D-aspartate (NMDA), α-amino-3-hydroxy-5-methyl-4-isoxazole propionic acid (AMPA), kainate receptors, and G-protein coupled receptor 35 (GPR35), and these receptors have a relevant role in pain processing and neuroinflammation [26]. A previous study suggests that KYNA has an anti-inflammatory effect on the TS [27]. In rats, KYNA had an analgesic effect in tail-flick test [28]. In an in vivo model of acetic acid-induced inflammatory pain, L-kynurenine, which is a precursor of KYNA, caused rise in the KYNA levels in the plasma and the central nervous system (CNS), thereby, was able to elicit anti-nociceptive effect [29].

Based on the results of clinical trials and animal experiments, trigeminal activation and sensitization occur during the migraine attack. Local administration of inflammatory soup (IS) on the dura mimics this process [30], which might be characterized by the alteration of selected molecular markers. On the other hand, since sumatriptan is effective in the acute treatment it may prevent these alterations and we wanted to compare its possible modulatory effects with a compound with a different pharmacological mechanism of action.

Thus, in our present study, we investigated the effect of IS induced dural inflammation on markers of the sensitization process in the trigemino-cervical complex, namely: CGRP, TRPV1, and nNOS. We also tested whether pretreatment with sumatriptan or KYNA has an effect on the IS induced changes.

## Materials and methods

### Animals

The procedures used in our study were approved by the Committee of the Animal Research of University of Szeged (I-74-49/2017) and the Scientific Ethics Committee for Animal Research of the Protection of Animals Advisory Board (XI./1098/2018) and followed the guidelines the Use of Animals in Research of the International Association for the Study of Pain and the directive of the European Parliament (2010/63/EU).

Seventy-two adult male Sprague-Dawley rats weighing 350–400 g were used. The animals were raised and maintained under standard laboratory conditions with tap water and regular rat chow available ad libitum on a 12 h dark-12 h light cycle.

### Drug administration

The animals were divided into two groups of 6 rats (*n* = 6 per group for 2.5 h and *n* = 6 per group for 4 h). The animals were deeply anesthetized with an intraperitoneal injection of 4% chloral hydrate (0.4 g/kg body weight, Sigma-Aldrich). The head of the animal was fixed in a stereotaxic frame and lidocaine (10 mg/ml, Egis) infiltration on the skull was used before the interventions. A handheld drill was used to make a window on the skull. The hole was made posterolaterally (5 mm, 3 mm) to the bregma, on the right side without penetrating the dura mater [31].

The animals in the first group called the placebo group, received synthetic interstitial fluid, (SIF, 135 mM NaCl, 5 mM KCl, 1 mM MgCl_2_, 5 mM CaCl_2_, 10 mM glucose in 10 mM HEPES buffer, pH 7.3). In the second group, we applied inflammatory soup (IS, 1 mM bradykinin, 1 mM serotonin, 1 mM histamine, 0.1 mM prostaglandin in 10 mM HEPES buffer, pH 5.0) on the dural surface. In the third and fourth groups, the animals received subcutaneous sumatriptan (0.6 mg/kg) 10 min before the SIF or IS treatment, while in the fifth and sixth groups received intraperitoneal KYNA (189.17 mg/kg) pretreatment one hour before treatment. Both pretreatment protocols were based on the pharmacological properties of the substances. Sumatriptan has a short half-life, its receptor binding is reversible and the onset of action is 10–15 min after administration. The half-life time of KYNA is about an hour. The dosage we used for both molecules was chosen based on previous reports [32–34]. Two and a half hours or four hours after the SIF or IS administration, the trigemino-cervical complex was processed for immunohistochemistry. Two survival times were used to examine changes over time.

### Immunohistochemistry

Two and a half hours or four hours after the SIF or IS administration, the rats were perfused transcardially with 50 ml phosphate-buffered saline (PBS, 0.1 M, pH 7.4), followed by 200 ml 4% paraformaldehyde in phosphate buffer under chloral hydrate anesthesia, and the trigemino-cervical complex was removed and postfixed overnight for immunohistochemistry in the same fixative. After cryoprotection, 30 μm cryostat sections were cut and serially collected in wells containing cold PBS. The free-floating sections were rinsed in PBS and immersed in 0.3% H_2_O_2_ in methanol (CGRP staining) or PBS (nNOS and TRPV1 staining) for 30 min. After several rinses in PBS containing 1% Triton X-100, sections were kept overnight at room temperature in anti- CGRP antibody (Sigma, C8198) at a dilution of 1:20000, or TRPV1 antibody (Santa Cruz, s.c.28759) at a dilution of 1:1000, or for two nights at 4 °C in anti-nNOS antibody (EuroProxima, 2263B220–1) at a dilution of 1:5000. The immunohistochemical reaction was visualized by the Vectastain Avidin-Biotin kit of Vector Laboratories (PK6101), and nickel ammonium sulfate-intensified 3,3′-diaminobenzidine. Control experiments included the omission of the primary antisera.

### Data evaluation

All evaluations were performed by an observer blind to the experimental groups. The photomicrographs of the stained sections of trigemino-cervical complex were taken using a Zeiss AxioImager microscope supplied with an AxioCam MRc Rev. 3 camera (Carl Zeiss Microscopy). The area covered by TRPV1-immunoreactive and CGRP-immunoreactive fibers was determined by Image Pro Plus 6.2® image analysis software (Media Cybernetics). After image acquisition, the laminae I–II in the dorsal horn were defined manually as areas of interest, and a threshold gray level was validated with the image analysis software. The program calculated the area innervated by the immunoreactive fibers as the number of pixels with densities above the threshold; the data were expressed as area fractions (%) of the corresponding immunolabelled structures. We measured the covered area by the CGRP and TRPV1 immunoreactive fibers and counted the nNOS immunoreactive cells in the area of the dorsal horn innervated by the ophthalmic nerve (V/1 area).

### Statistical analysis

The Shapiro-Wilk test was used to determine the distribution of data. As our data followed a normal distribution in each case, the differences among the groups and sides were examined with a mixed ANOVA model. The pairwise comparisons were performed by paired and independent samples t-tests with Sidak corrections. All statistical analyses were performed using SPSS version 24.0 (IBM Corporation). Values *p* < 0.05 were considered statistically significant. Our data are reported as means+SEM for all parameters and groups.

## Results

### Inflammatory soup and CGRP

**Fig. 1:**
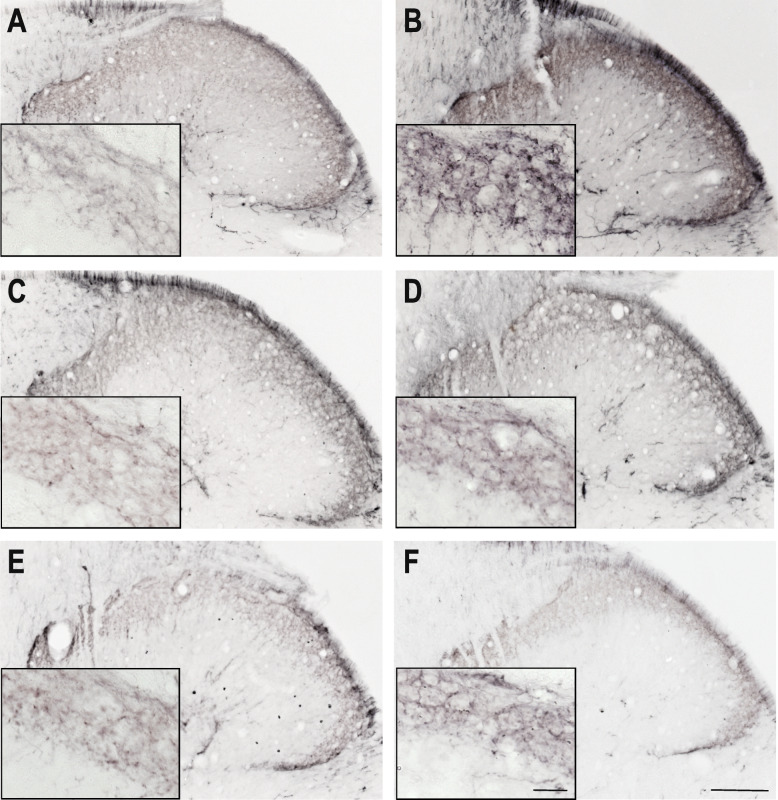
CGRP immunoreactivity 2.5 h after IS treatment Representative photomicrographs of the CGRP expression in the trigemino-cervical segments after 2.5 h. **a** – SIF, **b** – IS, **c** – SUMASIF, **d** – SUMAIS, **e** – KYNSIF, **f** – KYNIS. In the IS group, the CGRP staining was stronger than in the placebo group. Sumatriptan and kynurenic acid were able to attenuate this effect. SIF: synthetic interstitial fluid, IS: inflammatory soup, SUMASIF: sumatriptan + synthetic interstitial fluid, SUMAIS: sumatriptan + inflammatory soup, KYNSIF: kynurenic acid + synthetic interstitial fluid, KYNIS: kynurenic acid + inflammatory soup. Scale bars: 200 μm, 50 μm

**Fig. 2:**
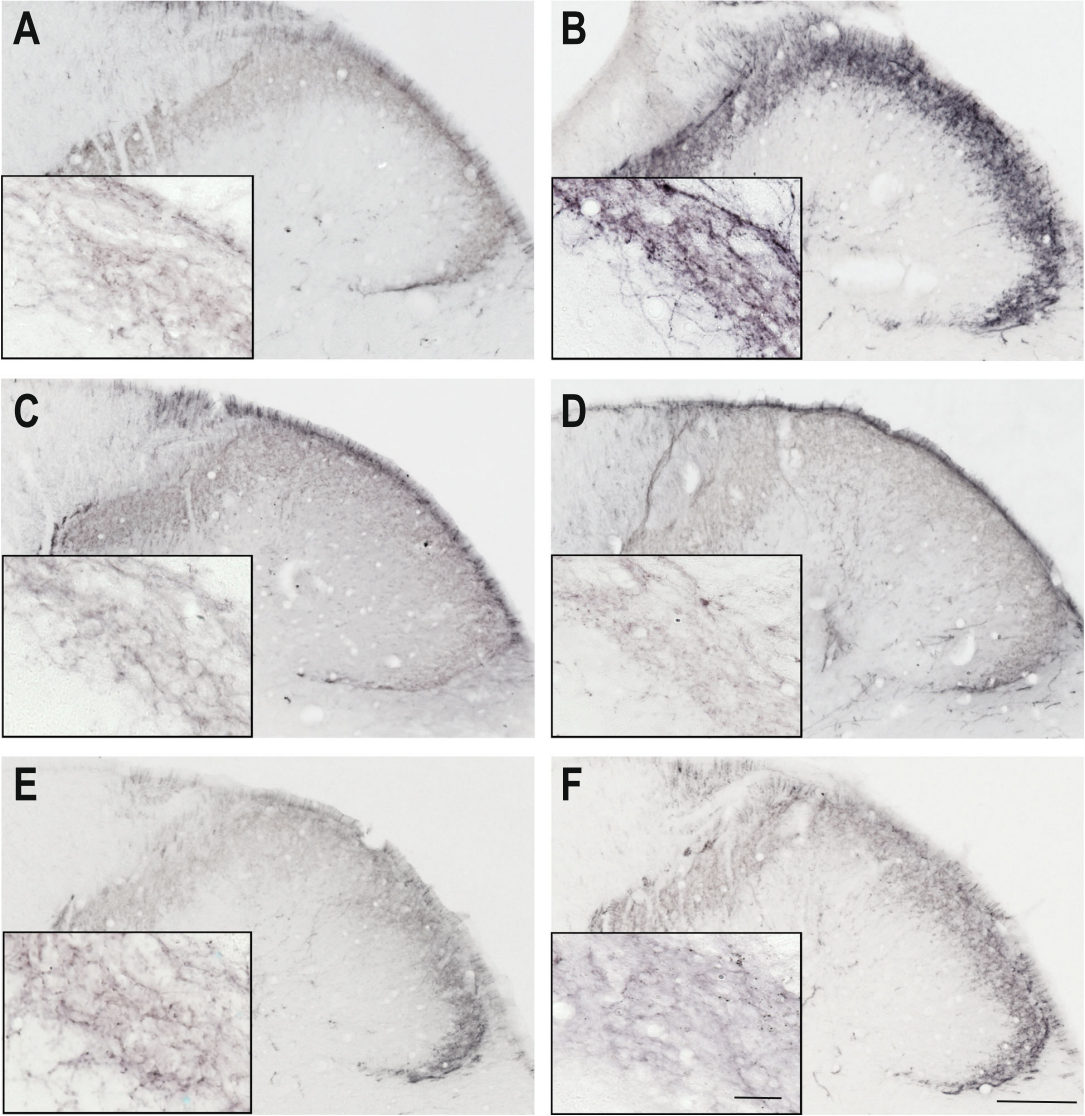
CGRP immunoreactivity 4 h after IS treatment Representative photomicrographs of the CGRP expression in the trigemino-cervical segments after 4 h. **a** – SIF, **b** – IS, **c** – SUMASIF, **d** – SUMAIS, **e** – KYNSIF, **f** – KYNIS. In the IS group, the CGRP staining was stronger than in the placebo group. Sumatriptan and kynurenic acid were able to attenuate this effect. SIF: synthetic interstitial fluid, IS: inflammatory soup, SUMASIF: sumatriptan + synthetic interstitial fluid, SUMAIS: sumatriptan + inflammatory soup, KYNSIF: kynurenic acid + synthetic interstitial fluid, KYNIS: kynurenic acid + inflammatory soup. Scale bars: 200 μm, 50 μm

**Fig. 3:**
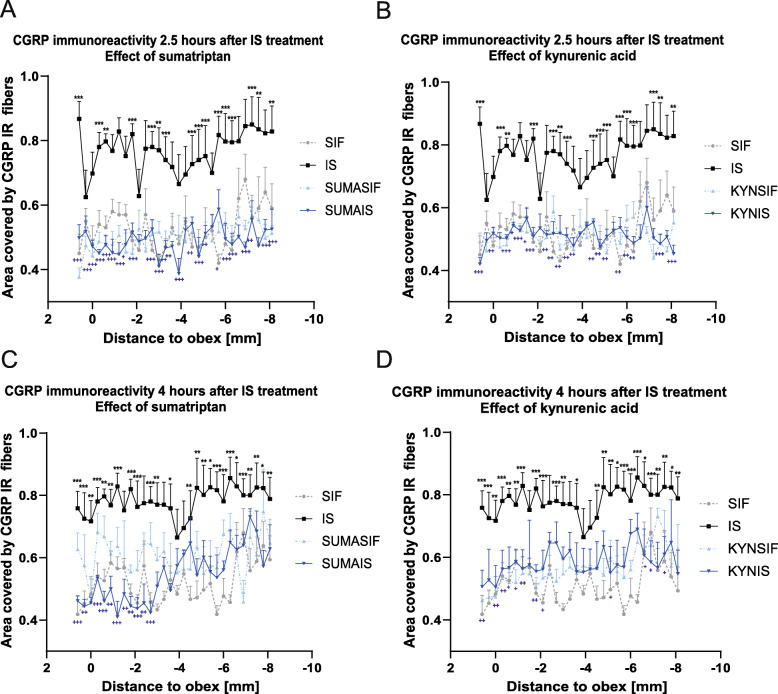
Statistical analysis of CGRP staining 2.5 and 4 h after IS treatment The quantitative analysis shows that in the IS group the area covered by fibers showing CGRP positivity is significantly higher than in the control group in both timepoints. **a** 2.5 h after IS treatment, sumatriptan was able to attenuate this effect in the V/1 area. **b** Similar to sumatriptan kynurenic acid weakened the effect of IS in the V/1 area. **c** 4 h after IS treatment, sumatriptan was able to mitigate this effect in the and V/1 area. **d** Kynurenic acid decreased the effect of IS in the V/1 area. *p < 0.05; **p < 0.01, ***p < 0.001 * means SIF-IS differences, + means IS-SUMA/KYNA. SIF: synthetic interstitial fluid, IS: inflammatory soup, SUMASIF: sumatriptan + synthetic interstitial fluid, SUMAIS: sumatriptan + inflammatory soup, KYNSIF: kynurenic acid + synthetic interstitial fluid, KYNIS: kynurenic acid + inflammatory soup

In the dorsal horn, CGRP immunoreactive (IR) axon fibers were distributed in the laminae I and II. IS treatment was able to increase the amount of the area covered by fibers showing CGRP positivity in both time points (Figs. 1, 2, and 3). Sumatriptan and KYNA pretreatments were able to attenuate this effect (Fig. 3). There was no relevant difference between the two time points in the area covered by fibers showing CGRP positivity.

### Inflammatory soup and TRPV1

**Fig. 4:**
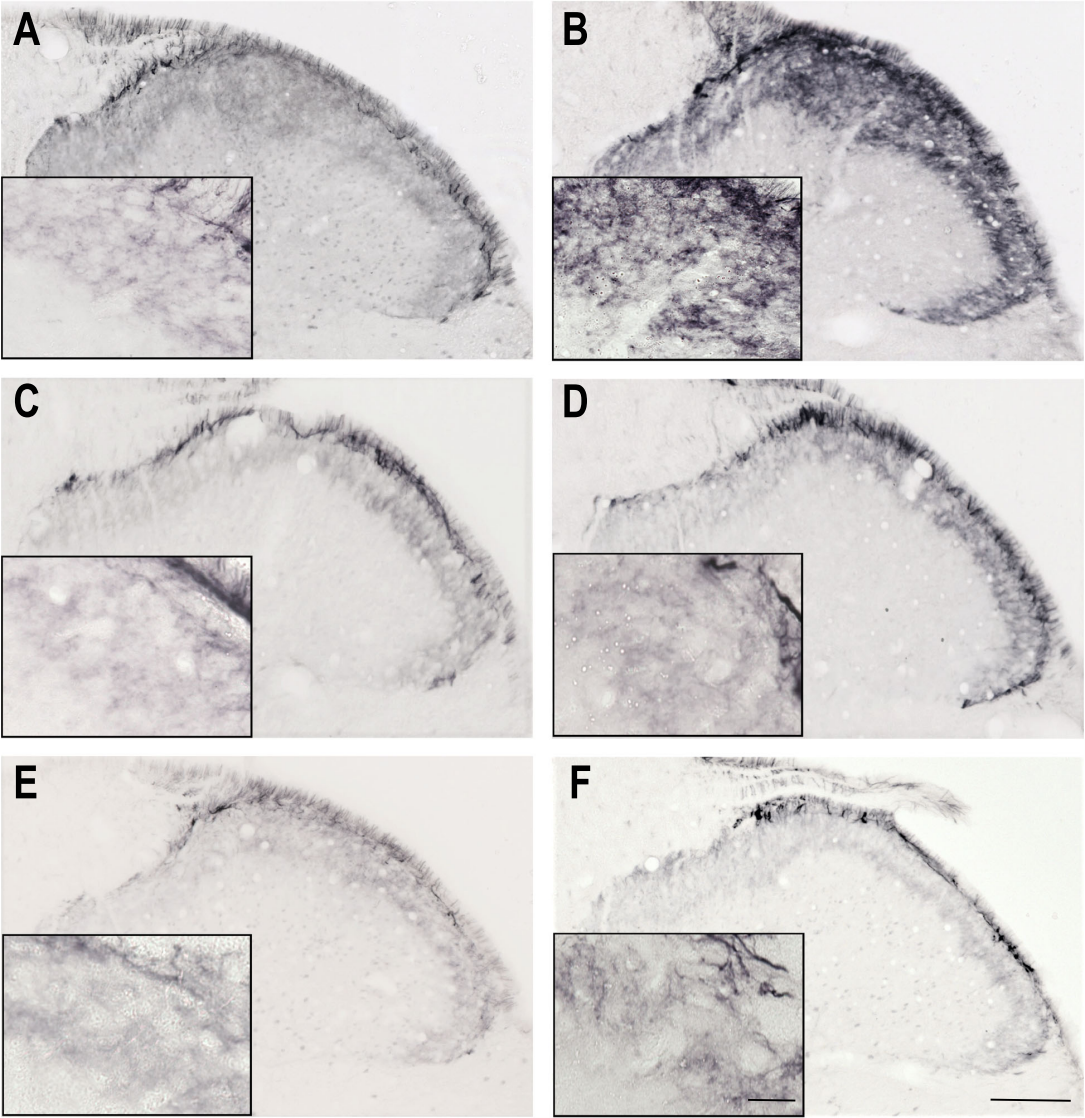
TRPV1 immunoreactivity 4 h after IS treatment Representative photomicrographs of the TRPV1 expression in the trigemino-cervical segments after 4 h. **a** – SIF, **b** – IS, **c** – SUMASIF, **d** – SUMAIS, **e** – KYNSIF, **f** – KYNIS. In the IS group, the area covered by TRPV1 was higher than in the placebo group. Sumatriptan and kynurenic acid were able to attenuate this effect. SIF: synthetic interstitial fluid, IS: inflammatory soup, SUMASIF: sumatriptan + synthetic interstitial fluid, SUMAIS: sumatriptan + inflammatory soup, KYNSIF: kynurenic acid + synthetic interstitial fluid, KYNIS: kynurenic acid + inflammatory soup. Scale bars: 200 μm, 50 μm

After 2.5 h there was no significant difference between the IS treated and placebo group (data not shown), but after 4 h we observed a significant increase in the amount of the TRPV1 IR fibers in the IS-treated group, compared to the placebo. Sumatriptan and KYNA pretreatments were able to mitigate the effect of the IS treatment (Fig. 4., Fig. 6.).

### Inflammatory soup and nNOS

**Fig. 5:**
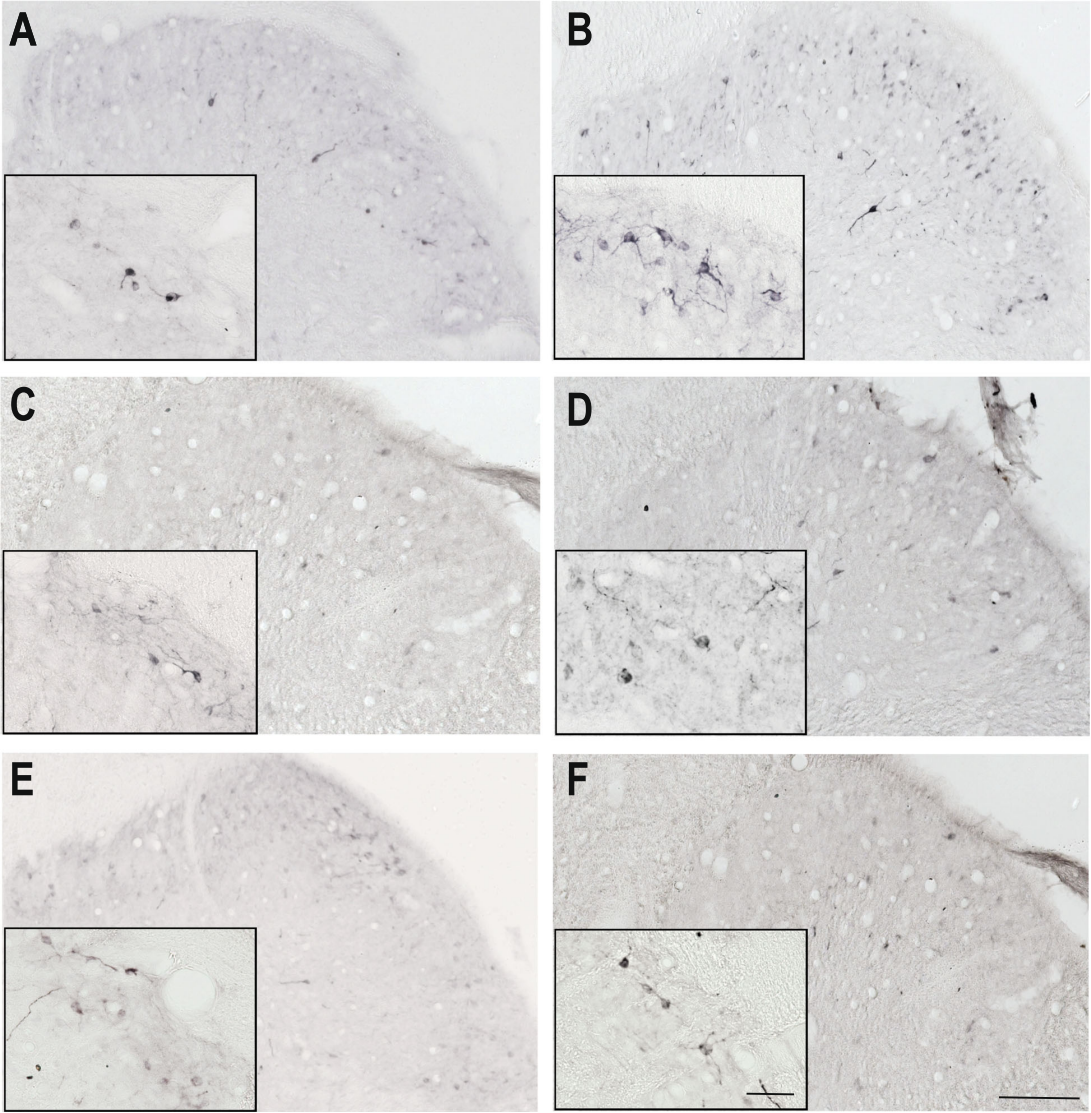
nNOS immunoreactivity 4 h after IS treatment Representative photomicrographs of the nNOS expression in the trigemino-cervical segments after 4 h. **a** – SIF, **b** – IS, **c** – SUMASIF, **d** – SUMAIS, **e** – KYNSIF, **f** – KYNIS. In the IS group, the number of nNOS-IR cells was increased compared to the SIF- treated group. Sumatriptan and kynurenic acid were able to mitigate this effect. SIF: synthetic interstitial fluid, IS: inflammatory soup, SUMASIF: sumatriptan + synthetic interstitial fluid, SUMAIS: sumatriptan + inflammatory soup, KYNSIF: kynurenic acid + synthetic interstitial fluid, KYNIS: kynurenic acid + inflammatory soup. Scale bars: 200 μm, 50 μm.

**Fig. 6:**
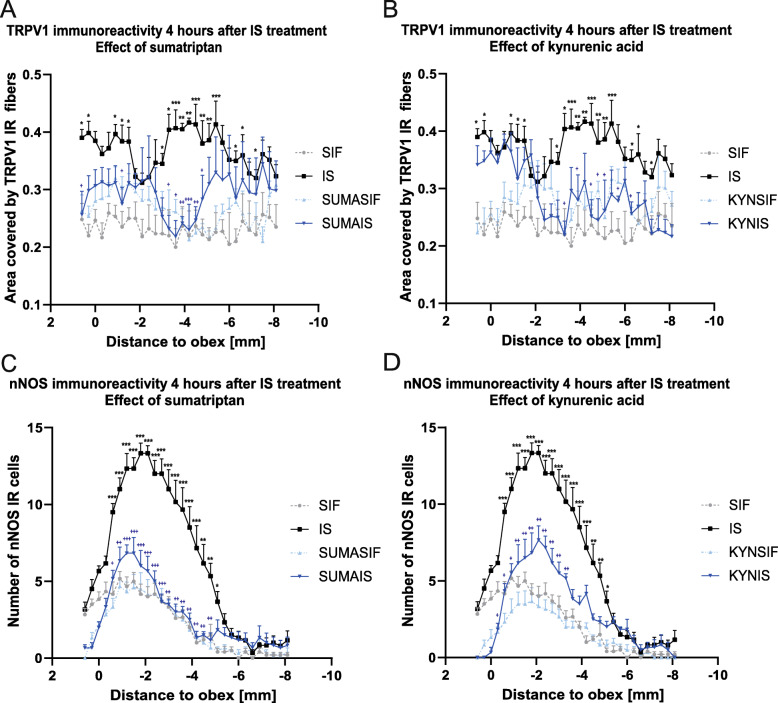
Statistical analysis of TRPV1 and nNOS staining 4 h after IS treatment The quantitative analysis shows that in the IS group the area covered by TRPV1 IR fibers and the number of nNOS IR cells is significantly higher than in the control group after 4 h. **a** Sumatriptan was able to attenuate the increase in TRPV1 IR fibers in the V/1 area. **b** Similar to sumatriptan kynurenic acid also mitigated the effect of IS in the V/1 area. **c** In the V/1 area sumatriptan was able to abolish the increase in nNOS IR cells. **d** Kynurenic acid also weakened the effect of IS in the V/1 area *p < 0.05; **p < 0.01, ***p < 0.001. * means SIF-IS differences, + means IS-SUMA/KYNA differences. SIF: synthetic interstitial fluid, IS: inflammatory soup, SUMASIF: sumatriptan + synthetic interstitial fluid, SUMAIS: sumatriptan + inflammatory soup, KYNSIF: kynurenic acid + synthetic interstitial fluid, KYNIS: kynurenic acid + inflammatory soup

A significant increase of nNOS was observed only after 4 h in the IS treated group compared to the placebo group in the V/1 area. Sumatriptan and KYNA pretreatments were able to modulate the effect of IS administration (Fig. 5., Fig. 6.).

## Discussion

The activation and sensitization of the TS are essential hallmarks of migraine pathomechanism. In our rat model, the topically applied IS activated the trigeminovascular system [35, 36], and raised the levels of all the three selected markers in the TNC area. Although other pathways, neurotransmitters (e.g. glutamate, 5HT, prostaglandins) and mechanisms might be involved, the release of neuropeptides from the activated peripheral nociceptive terminals may contribute to the development of neurogenic inflammation in this case [3]. These peptides lead to a cascade of inflammatory tissue responses including vasodilation, plasma protein extravasation, and degranulation of mast cells [3], at least in rats.

In our study, as early as two and a half hours after administration, the IS was able to increase the area covered by fibers showing CGRP positivity in the dorsal horn of the cervical spinal cord. The cranial dura mater is densely innervated by CGRP IR fibers [37] thus the increased CGRP level might represent enhanced activation of the primary afferents, which may also be associated with increased CGRP release from the terminals [9] possibly causing a globally higher turnover e.g. intensive synthesis reflected by higher CGRP expression at the terminals [38]. It has been also shown, that intracisternal IS can raise the CGRP concentration in the jugular vein which also reflects release from the nerve endings [39]. This phenomenon might contribute to the activation and sensitization of primary and secondary nociceptors in the TS via the release of numerous pro-inflammatory agents (e.g. cytokines), which can stimulate the nociceptors [40].

In our experiment, IS was able to significantly increase the amount of TRPV1 IR fibers in the dorsal horn after 4 h. Amaya and colleagues described that TRPV1 expression is showing up-regulation in DRG neurons after local inflammation in rats [41]. TRPV1 activation by high temperature or capsaicin allows the entry of Ca^2+^, leading to the release of neuropeptides [42, 43], which are able to influence the development of edema and neurogenic inflammation [44, 45]. In another study, after CFA-induced inflammation, increased TRPV1 expression was observed in the digital nerves of the inflamed hindpaw [46]. Pharmacologic studies have also shown that TRPV1 is an essential component of the cellular signaling mechanisms through which injury produces thermal hyperalgesia and pain hypersensitivity [47].

TRPV1 is present in the human TG [48] and trigeminal afferents, which innervate the dura mater [49], and these nerve fibers also contain CGRP [50]. TRPV1 expression is also upregulated in painful inflammatory conditions in humans [51]. In chronic migraine patients, intranasal capsaicin was able to mitigate the migraine pain [52] and TRPV1 agonists might be effective most likely due to desensitization in the acute treatment of migraine [53] as well. Taken together these data point to the fact, that TRPV1 is involved in the trigeminovascular activation and sensitization both in animal models and humans.

In our study, IS significantly enhanced the number of nNOS IR cells in the dorsal horn after 4 h due to the activation of primary trigeminal nociceptors conveyed to the second-order neurons [54]. NO donors cause an increase and release of CGRP at the TG and TNC, and NO donors lead to a delayed enhancement of nNOS in the latter [55]. Moreover, bradykinin and histamine trigger NO release from vascular endothelial cells in vitro, suggesting a strong interaction between NO and inflammation [56]. An increase in NO production may contribute to an amplifying process in the meninges, which involves the release of CGRP and possibly prostaglandins and other mediators leading to rapid vasodilatation [19, 57]. The latter can lead to the activation of primary afferent neurons and CGRP release, activating satellite glial cells that release NO, which can induce nNOS [58]. In this context nNOS is can be considered as a significant marker of the sensitization process of the TS in animals.

Interestingly, the increase of TRPV1 and nNOS levels are observed later compared to the changes of CGRP reflecting, that the changes of the latter are more likely related to the activation of the primary trigeminal nociceptors whereas TRPV1 and nNOS, which are more likely involved in the sensitization, show a delayed pattern of enhancement.

In our study, sumatriptan was able to modulate the increase of CGRP levels and the TRPV1 activity probably through 5-HT_1B/1D_ receptors. This is in line with previous results showing, that CGRP and TRPV1 are co-localized with 5-HT_1B/1D_ receptors in trigeminal neurons [59] and sumatriptan presynaptically inhibits the release of nociceptive neuropeptides (e.g. CGRP) from primary afferents [60] and most of the effects of TRPV1 receptors are mediated through CGRP, which is released after TRPV1 activation [61] so 5-HT might have a role in modulation of TRPV1 function too. This is paralleled with the observations, where sumatriptan mitigated the TRPV1 activity after the intracisternal application of IS [62].

In our experiment, sumatriptan also prevented the increase in the number of nNOS IR cells in the rat TNC after 4 h suggesting an important involvement of 5-HT_1B/1D_ receptors in the sensitization process in the TS. On the periphery, sumatriptan inhibits presynaptically the release of vasoactive peptides from primary afferents and impairs the development of neurogenic inflammation [25]. Sumatriptan prevented the increased NOS production in the brainstem after intracisternal carrageenan injection [63]. In humans, NTG-induced headache has been reported to respond to sumatriptan as well [64]. Taken together, these results suggest that 5-HT_1B/1D_ agonism can inhibit IS-induced activation and sensitization present in dural inflammatory process.

Compared to sumatriptan**,** KYNA had a similar effect on the examined markers in our experimental setting and this phenomenon may be mediated through several different receptors. First KYNA is an endogenous, non-selective ionotropic glutamate receptor antagonist, which acts on the non-competitive glycine site of NMDA receptors and it is also a GPR35 ligand [26]. Currently, the antagonist effect of KYNA on the α7-nicotinic acetylcholine receptor (nAChR) is contested [65].

Three hours after the local IS treatment of the dura, higher glutamate levels can be detected in the TNC [57]. In addition to the NMDA receptors, both AMPA, kainate, and metabotropic receptors are found in the TNC [66] and it has been shown, that the antagonists of non-NMDA glutamate receptors also can inhibit the activation of secondary nociceptive neurons [67]. AMPA receptors can modulate c-fos expression and possibly the neurotransmission in the TS [68] and in a peripheral pain model, activation of the kainate receptors resulted in the appearance of mechanical, thermal hyperalgesia, and allodynia [67]. In the TNC, CGRP can stimulate glutamate expression and that can be inhibited by 5-HT_1B/1D_ receptor agonists [69]. Hence, the relationship between the two systems can be assumed.

In humans, painful stimulation leads to an increase in glutamate concentration in the trigemino-cervical complex [70] and glutamate levels are elevated both ictally and interictally in migraine sufferers [71]. Based on observations in animals and humans, we believe that among others the modulation of glutamatergic neurotransmission is the key event here mitigating CGRP changes.

TRPV1 and NMDAR are co-expressed in the TG [72]. In a mechanical hyperalgesia test of rats, it was found, that NMDAR and TRPV1 functionally interact probably via the calcium/calmodulin-dependent protein kinase type II (CaMKII) and protein kinase C signaling cascades in rat trigeminal sensory neurons and this interaction has a role in the development of mechanical hyperalgesia [72]. GPR35 and TRPV1 co-localize in small- and medium diameter DRG neurons. GPR35 may regulate TRPV1 channel activity by modulating cyclic adenosine monophosphate /protein kinase A pathway [73].

KYNA pretreatment also modulated the IS induced nNOS expression in our animal model. This effect might be related to the anti-glutamatergic effect of KYNA, mainly on the NMDA receptors, which activation is associated with NO production in the spinal trigeminal nucleus [74]. Furthermore, Cosi and colleagues described that elevation of KYNA concentration in the brain could decrease the extracellular glutamate levels in the nervous tissue and reduce inflammatory pain [75]. Another possible explanation for the peripheral effects of KYNA is that it binds to GPR35, which receptor is present in the DRG [76] and KYNA can inhibit adenylate cyclase activity there via G-protein-dependent mechanisms [73] which might interact with nNOS [77, 78]. It has been also reported that abnormalities of the kynurenine pathway are associated with headache disorders e.g. there is evidence that serum kynurenic acid levels decrease during cluster headache and chronic migraine [79–81].

In the present study, sumatriptan and KYNA were similarly effective mitigating the effects of the IS model. They were likely to exert their effects through different receptors/pathways involved in the activation of the trigeminovascular system pointing to different sites of possible pharmacological modulation during this process.

## Conclusion

In our experiment, IS was able to cause sterile neurogenic inflammation in the dura mater. As a consequence of inflammation, changes occur in CGRP, TRPV1, and nNOS levels, which indicates activation and sensitization. Sumatriptan probably acted through the 5-HT_1B/1D_ receptors to reduce the expression of the activation and sensitization markers in the TNC by direct (presynaptic) and indirect (lowered dural inflammation) effects on the periphery. In our present study, KYNA possibly acted primarily on peripheral trigeminal nociceptors and secondary sensory neurons and was able to mitigate the activation of these markers in the TNC predominantly through the inhibition of the glutamate system and thereby blocking the sensitization processes, which is important in primary headaches. These findings can help us to understand the pathomechanism processes in migraine.

## Data Availability

The data that support the findings of this study are available from the corresponding author upon request.

## Abbreviations

AMPA: α-amino-3-hydroxy-5-methyl-4-isoxazole propionic acid
CaMKII: calcium/calmodulin-dependent protein kinase type II
CGRP: calcitonin-gene related peptide
CNS: central nervous system
IR: immunoreactive
IS: inflammatory soup
DRG: dorsal root ganglion
KYNA: kynurenic acid
nAChR: α7-nicotinic acetylcholine receptor
nNOS: neuronal nitric oxide synthase
NMDA: N-methyl-D-aspartate
NO: nitric-oxide
NTG: nitroglycerin
PBS: phosphate-buffered saline
PBS-T: PBS containing 1% Triton-X-100
PFA: paraformaldehyde
SIF: synthetic interstitial fluid
TG: trigeminal ganglion
TNC: caudal trigeminal nucleus
TRPV1: transient receptor potential vanilloid-1 receptor
TS: trigeminal system
V/1: ophthalmic nerve
5-HT: 5-hydroxytryptamine
